# Continuous debridement combined with short-term posaconazole therapy for cutaneous mucormycosis caused by *Rhizopus oryzae* infection secondary to acute myeloid leukemia: a case report

**DOI:** 10.3389/fmed.2024.1448147

**Published:** 2024-10-23

**Authors:** Fengming Wang, Jv Li, Yilian Xie, Jiayuan Ye

**Affiliations:** ^1^Department of Hematopathology, Shangyu People’s Hospital of Shaoxing, Shaoxing, Zhejiang, China; ^2^Department of Pediatrics, Shangyu People’s Hospital of Shaoxing, Shaoxing, Zhejiang, China; ^3^Department of Infectious Diseases, The First Affiliated Hospital of Ningbo University, Ningbo, Zhejiang, China; ^4^Department of Infectious Diseases, Shangyu People’s Hospital of Shaoxing, Shaoxing, Zhejiang, China

**Keywords:** cutaneous mucormycosis, lower limb infection, acute myeloid leukemia, posaconazole, case report

## Abstract

Cutaneous mucormycosis is a rare fungal infection marked by skin abscesses, swelling, necrosis, dry ulcers, and eschars. Though less fatal compared to other mucormycosis forms, delayed diagnosis and treatment in immunocompromised patients can cause the infection to spread to vital organs, becoming life-threatening. We report a case of lower extremity cutaneous mucormycosis secondary to acute myeloid leukemia, successfully managed with sustained surgical debridement and short-term oral posaconazole. This case highlights the effectiveness of surgical debridement and the potential for short-course antifungal therapy in managing cutaneous mucormycosis.

## 1 Introduction

Mucormycosis primarily infects patients with severe underlying conditions that compromise the immune system. These conditions include hematologic malignancies, solid organ or hematopoietic stem cell transplantation, uncontrolled diabetes mellitus, and ketoacidosis (1–4). Cutaneous mucormycosis typically occurs when the skin barrier is disrupted due to various traumas, surgeries, or burns, allowing fungal spores to directly inoculate the damaged skin and potentially lead to dissemination to various organs.

Mucormycosis is a rare but highly fatal disease. The mortality rate ranges from 20 to 50% in cases of localized infection. In cases of disseminated mucormycosis, the mortality rate can reach as high as 70 to 90%. These statistics emphasize the importance of early diagnosis and treatment to reduce patient mortality (4–6). China and India, as populous countries, have relatively high incidences of mucormycosis, estimated to be between 36 and 40% (1). A retrospective analysis of mucormycosis infection data in China from January 2001 to July 2020 reported an overall mortality rate of 37.2%. Specifically, the highest mortality was observed in cases of disseminated mucormycosis, with a rate of approximately 63.9%. In addition, rhino-orbital cerebral mucormycosis (ROCM) had a mortality rate of 54.4%, whereas cutaneous mucormycosis had a significantly lower mortality rate of approximately 17.1% (7).

In this study, we reported a case of acute myeloid leukemia (AML) patient who developed a localized skin infection during chemotherapy, later confirmed to be caused by *Rhizopus oryzae*. We treated the patient with continuous surgical debridement combined with short-term posaconazole therapy.

## 2 Case report

**Figure 1:**
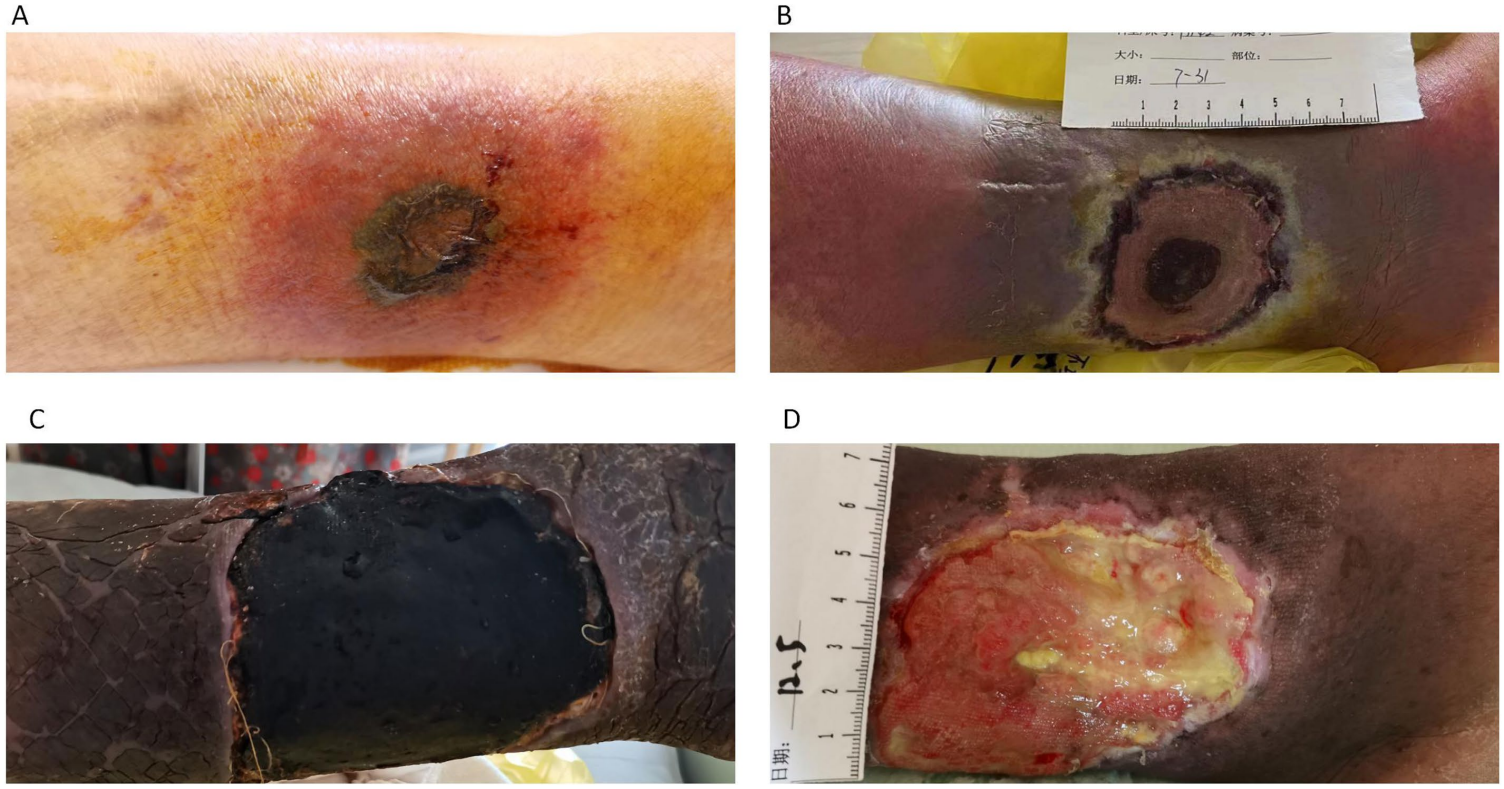
**(A)** Infection site at the time of patient admission. **(B)** Lesion enlargement after 10 days of standard antibacterial treatment. **(C)** Debridement and bone cement implantation. **(D)** Clean wound with gradual recovery.

The patient is an 80-year-old elderly male living in China, who was admitted to the Hematology Department of Shaoxing Shangyu People’s Hospital due to fever and skin breakdown for 3 days. On July 18, 2023, the patient presented with a 1 cm × 1 cm wound with surface breakdown and slight tenderness and redness on the left lower leg (Figure 1A). The border of the skin lesion was clear, accompanied by difficulty in walking. The patient had not sought medical attention at any other healthcare institution prior to admission. Upon admission on July 21, standard empirical antimicrobial therapy showed poor efficacy (Figure 1B). Subsequent fungal culture and fluorescent staining suggested mucormycosis and mass spectrometry identified the pathogen as *Rhizopus oryzae*. The patient underwent an expansion of the debridement area and negative pressure drainage, along with adjunctive treatment with enteric-coated posaconazole tablets. The patient’s fever gradually subsided, and debridement was continued, followed by antibiotic cement implantation surgery (Figure 1C). After continuous debridement for 4 months, by December, the wound had further reduced in size and began to heal (Figure 1D). The patient was discharged after a reassessment of blood routine and other indicators.

### 2.1 Admission physical examination

A 1 cm × 1 cm area of purplish-red discoloration was presented on the left lower limb. The lesion had a slight central breakdown covered by a purplish-red crust and exhibited clear borders. The surrounding area appeared red, and there was noticeable tenderness upon palpation.

### 2.2 Medical history

The patient was diagnosed with acute myeloid leukemia (AML) M5 subtype with FLT3 gene mutation in March 2023. Targeted therapy was initiated at the Hematology Department of Shaoxing Shangyu People’s Hospital. The initial treatment regimen consisted of BCL-2 inhibitor at a dose of 200 mg, escalated to 600 mg quaque die (qd), along with azacitidine 135 mg from day 1 to day 7. However, treatment was interrupted due to hepatic impairment. Subsequently, the patient was switched to a combination therapy of BCL-2 inhibitor 600 mg qd plus gilteritinib 120 mg qd. During this period, hemolysis occurred, which was managed with steroid therapy. Upon alleviation of symptoms, the patient was discharged and continued targeted therapy along with oral prednisone acetate 10 mg peros ter in die (po, tid) to control hemolysis. On July 29th, bone marrow examination revealed phenotypic abnormalities, with immature cells of myeloid lineage comprising 0.2% of nucleated cells and a significantly increased proportion of nucleated red blood cells, accompanied by partial loss of CD36 expression in some cells.

### 2.3 Laboratory findings

**Figure 2:**
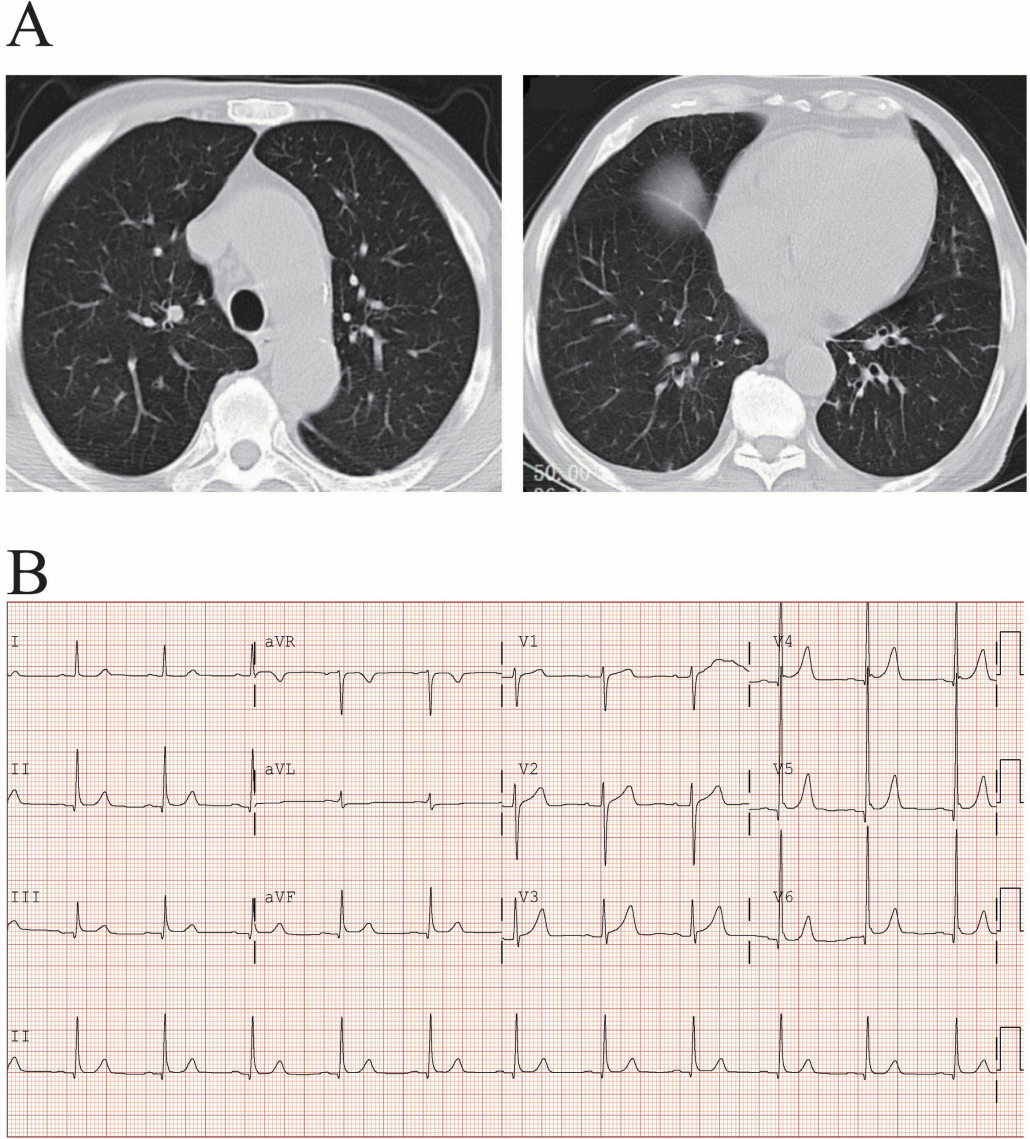
Chest computed tomography **(A)** and electrocardiogram **(B)** showed no abnormalities.

The physical examination data and laboratory test results from the day of admission are detailed in Table 1. Chest Computed Tomography (CT), and electrocardiogram showed no abnormalities (Figure 2).

**Table 1:** The physical examination data and laboratory test results.

Tests	Result	Reference range	Interpretation
Patient’s vitals			
BP (mmHg)	120/70	<120/80	Normal
HR (beats/min)	85	60–100	Normal
RR (breaths/min)	17	Dec-20	Normal
T (°C)	38.5	37	High
CBC test			
WBC (10*9/L)	4.7	3.5–9.5	Normal
NEU (10*9/L)	1.02	1.8–6.3	Low
LYM (10*9/L)	0.64	1.1–3.2	Normal
RBC (10*9/L)	1.93	4.3–5.8	Low
HGB (g/L)	62	130–175	Low
MCV (fl)	90.5	82–100	Normal
PLT (10*9/L)	10	125–350	Low
CRP (mg/L)	2.2	<8	Normal
Laboratory chemistry			
ALT (U/L)	14.4	Sep-50	Normal
AST (U/L)	40.3	15–40	High
TBIL (μmol/L)	27	4.5–22	High
DBIL (μmol/L)	10.3	0–6	High
IBIL (μmol/L)	16.7	1.5–14	High
Immune indicators			
1,3-beta-D-glucan (pg/mL)	<37.5	<37.5	Negative
Galactomannan	0.7	<0.5	Positive

BP, blood pressure; HR, heart rate; RR, respiration rate; T, temperature; CBC, complete blood count; WBC, white blood cells; NEU, neutrophil; EOS, eosinophil; LYM, lymphocyte; RBC, red blood cells; HGB, hemoglobin; MCV, mean corpuscular volume; PLT, platelet; CRP, C-reactive protein; ALT, alanine aminotransferase; AST, aspartate aminotransferase; TBIL, total bilirubin; DBIL, direct bilirubin; IBIL, indirect bilirubin.

### 2.4 Fungal culture

**Figure 3:**
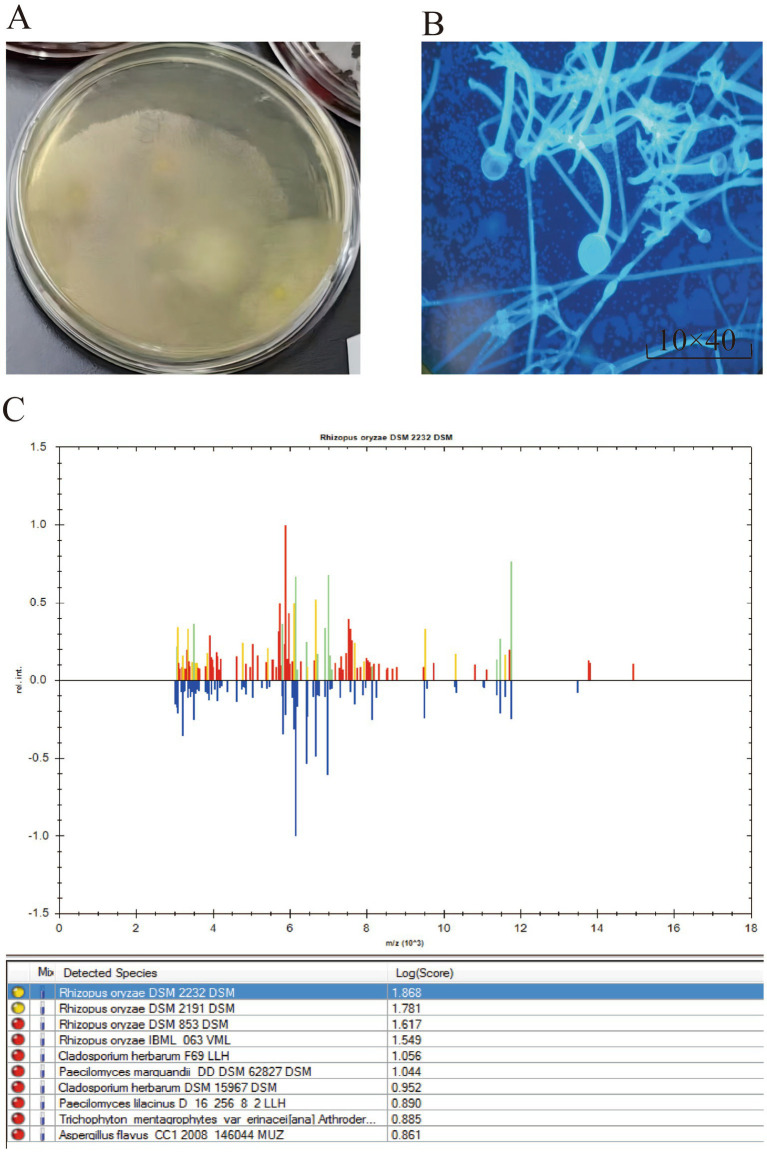
**(A)** White, woolly colonies observed after 72 h of microbial culture. **(B)** Thick hyphae without septation, presence of false roots and creeping mycelium, large-sized sporangia, spherical sporangiospores, and elliptical sporangiophores. **(C)** The results of MALDI-TOF Mass Spectrometry Detection indicate that the microorganism is *Rhizopus oryzae*.

Tissue specimens were inoculated onto Columbia blood agar, chocolate agar, and Sabouraud agar plates. Rapid growth of white, fluffy colonies was observed after 72 h of incubation at 37°C in a 5% CO_2_ atmosphere (Figure 3A).

### 2.5 Fluorescent staining method and results

The glass slide was placed horizontally, and an appropriate amount of the sample was applied. A drop of staining solution (composed of disodium hydrogen phosphate, sodium dihydrogen phosphate, potassium chloride, *β*-D-glucan binding protein, fluorescein, counterstain, and purified water) was added directly onto the sample, ensuring that the solution covered or immersed the entire sample. Staining was allowed to proceed for several minutes. After covering with a coverslip and removing excess staining solution, the sample was observed under a fluorescence microscope. The microscopy revealed thick, non-septate hyphae with visible rhizoids and stolons. The sporangia were large, with round sporangiospores and an ovoid columella, consistent with the typical morphological characteristics of Mucorales (Figure 3B).

BD Bruker Time-of-Flight MALDI-TOF Mass Spectrometry Detection Method and Results: The following steps were carried out for microbial sample preparation: (1) The microbial sample was collected. (2) 300 μL of water was added to the sample and mixed thoroughly. (3) 900 μL of ethanol was added and mixed. (4) An appropriate amount (10–50 μL) of 70% formic acid solution was added and mixed. (5) An appropriate amount (10–50 μL) of acetonitrile was added, mixed, and the mixture was centrifuged. (6) 1 μL of the supernatant was applied to the MALDI target plate and air-dried. Subsequently, 1 μL of matrix was added, air-dried, and the sample was analyzed by MALDI-TOF mass spectrometry, identifying the result as *Rhizopus oryzae* (Figure 3C).

### 2.6 Treatment

Upon admission, the patient received oral linezolid, (600 mg po Q12H) in combination with intravenous piperacillin-tazobactam (450 mg Intravenous Q8H) as routine empirical antimicrobial therapy. After 3 days, the area of erythema expanded to 3 cm × 3 cm. Debridement and cultures were performed, followed by simple debridement and topical application of (Recombinant Bovine Basic Fibroblast Growth Factor For External Use and Sulfanilamidopyrimidine). The patient continued to have persistent fever, accompanied by further enlargement of the wound to 7 cm × 7 cm. On July 30th, fungal culture combined with fluorescent staining reported the presence of mold, which was identified as *Rhizopus oryzae* based on species identification. Linezolid, and piperacillin-tazobactam were discontinued, and the wound was expanded for debridement along with negative pressure drainage. Posaconazole enteric-coated tablets 400 mg po, bis in die (bid) were administered as antifungal therapy for 1 week. Due to financial constraints, the antifungal medication had to be temporarily suspended. The patient’s fever subsided, but due to poor wound healing, after continuous debridement for 1 month, antibiotic bone cement implantation was performed. The wound began to shrink, and the patient was able to ambulate. Continuous debridement was continued for 3 months, and by December, the wound had further reduced in size, demonstrating recovery (Figure 1D). On December 18th, follow-up blood tests including complete blood count and highly sensitive C-reactive protein (CRP) showed: White blood cells (WBC) 3.8*10^9/L, neutrophil% 20.1, lymphocyte% 77.3, Red blood cells (RBC) 1.76*10^12/L, hemoglobin 51 g/L, hematocrit 15.6%, mean corpuscular volume 88.7 fl, platelet count 55*10^9/L, highly sensitive CRP 36.5 mg/L. The patient was discharged. Subsequently, the patient was readmitted due to fatigue in January 2024. At that time, Complete blood count (CBC) showed WBC 36.5*10^9/L, RBC 2.14*10^12/L, hemoglobin 68 g/L, platelet count 27*10^9/L, and platelet distribution width 17.3%. On January 4th, the bone marrow puncture examination was completed, revealing phenotypic abnormalities with 3.8% of nucleated cells being immature myeloid cells and a significantly increased proportion of nucleated red blood cells, accompanied by partial loss of CD36 expression in some cells. Routine bone marrow examination suggested 6.5% primitive granulocytes, indicative of relapsed acute myeloid leukemia. Due to poor response to chemotherapy, the patient eventually succumbed.

## 3 Discussion

Mucormycosis is an invasive fungal disease caused by Mucorales infections, with Rhizopus being the most common pathogenic genus, followed by Mucor, Rhizomucor, and Lichtheimia (4, 8). Human infections mainly occur through inhalation of fungal spores, although occasionally they can also result from ingestion of contaminated food or through skin wounds (9, 10). The risk of mucormycosis significantly increases in individuals undergoing high-dose chemotherapy, experiencing granulocytopenia, or receiving immunosuppressive therapy, particularly in patients with hematological disorders (11).

Dermatophytes are morphologically characterized by wide, irregular, unsegmented, or occasionally segmented hyphae with right-angled branching. Cutaneous dermatophytosis is primarily categorized into two clinical types: acute necrotizing and subacute or chronic dermatophytosis. The acute form manifests as erythema, swelling, plaques, pustules, ulcers, necrosis, and eschars (12). Conversely, the subacute or chronic form typically develops following minor trauma or insect bites (13), predominantly affecting exposed areas of the face and extremities. It is characterized by skin plaques, swelling, and progressive ulceration. Hematogenous dissemination due to necrosis in cutaneous dermatophytosis is common and carries a grave prognosis. Despite having the lowest mortality rate among all forms of dermatophytosis at 17.1% (7), this rate increases to 31% in cases of deep-seated infections (14). Furthermore, it has been reported that in the presence of underlying risk factors, 3% of cases progress to disseminated infections with mortality rates escalating to 83–94% (4, 15).

Hematology patients are prone to opportunistic infections, especially mucormycosis, which may be related to the decreased immune function of patients with hematologic disorders themselves. This is further exacerbated by the prolonged use of chemotherapy, glucocorticoids, immunosuppressants, and broad-spectrum antibiotics, leading to a further decline in the body’s immune function. Research suggests that neutrophils and macrophages are the primary mechanisms by which the host defends against mucormycosis infection. Neutrophils can destroy fungal hyphae, while macrophages can kill fungal spores within cells through oxidative mechanisms (16). Additionally, glucocorticoid therapy can decrease macrophage function, weakening their ability to clear fungal spores (17). Furthermore, studies have found that the growth of mucormycetes requires involvement in iron metabolism. Hematologic patients often experience iron overload due to long-term blood transfusions, which increase the abundance of free iron, favoring fungal proliferation. Moreover, the use of iron chelators also increases the risk of mucormycosis infection in patients (18).

The European Confederation of Medical Mycology (ECMM) guidelines strongly recommend surgical intervention as the first-line treatment when mucormycosis is highly suspected. Additionally, high-dose liposomal amphotericin B is advised for initial therapy, while isavuconazole and posaconazole are preferred for salvage therapy. Currently, the exact duration of treatment for mucormycosis is unclear, and there is no strong evidence supporting combination antifungal therapy. The guidelines emphasize the priority of surgical treatment, with repeated debridement of the lesion if necessary (19).

Regarding surgical treatment, an article suggests that using antifungal agents alone or conservative debridement alone cannot prevent the progression of mucormycosis infection (20). Furthermore, certain types of mucormycetes demonstrate high sensitivity to antifungal agents *in vitro* but have poor efficacy *in vivo* (21). Therefore, surgical debridement can effectively improve the treatment outcomes of mucormycosis infections. Case reports indicate that patients with cutaneous mucormycosis infections have achieved better treatment outcomes and shorter treatment durations by combining surgical closure, skin grafting, continuous negative pressure suction, and drug therapy (22). During debridement surgery, to prevent the implantation and dissemination of mucormycetes, it is recommended to confirm through pathological examination of debrided tissues that the margins of the wound are fungal-negative. Local recurrence is common within 2 days postoperatively, so it is necessary to observe and evaluate the wound daily after debridement, with most cases requiring continuous debridement (23).

Based on the recommendations of the guidelines and successful cases, we immediately performed thorough debridement and initiated systemic antifungal therapy with posaconazole upon diagnosing mucormycosis in our patient. Despite only using posaconazole for 1 week due to economic constraints, we achieved successful treatment outcomes with continuous thorough debridement. Currently, there are no specific recommendations regarding the duration of antifungal therapy. Therefore, in the treatment of cutaneous mucormycosis, combining surgical intervention with short-term antifungal therapy may be a preferable option.

In conclusion, cutaneous mucormycosis is a rare but serious complication in hematologic malignancies, with no standardized treatment guidelines currently available. This case study demonstrates the successful management of cutaneous mucormycosis through continuous surgical debridement combined with short-term antifungal therapy. The findings underscore the potential benefits of prioritizing surgical intervention while reducing the duration of systemic antifungal treatment. This approach not only enhances treatment efficacy but also offers a more rational and cost-effective strategy for managing cutaneous mucormycosis, which could inform future treatment protocols.

## Data Availability

The raw data supporting the conclusions of this article will be made available by the authors, without undue reservation.
